# A view to a kill? – Ambient bacterial load of frames and lenses of spectacles and evaluation of different cleaning methods

**DOI:** 10.1371/journal.pone.0207238

**Published:** 2018-11-28

**Authors:** Birgit Fritz, Anne Jenner, Siegfried Wahl, Christian Lappe, Achim Zehender, Christian Horn, Frithjof Blessing, Matthias Kohl, Focke Ziemssen, Markus Egert

**Affiliations:** 1 Faculty of Medical and Life Sciences, Institute of Precision Medicine, Microbiology and Hygiene Group, Furtwangen University, Villingen-Schwenningen, Germany; 2 Carl Zeiss Vision International GmbH, Aalen, Germany; 3 Institute for Ophthalmic Research, University of Tuebingen, Tuebingen, Germany; 4 Institute for Laboratory Medicine, Singen, Germany; 5 Faculty of Medical and Life Sciences, Institute of Precision Medicine, Group for Statistics in Biology and Medicine, Furtwangen University, Villingen-Schwenningen, Germany; 6 Center for Ophthalmology, Eberhard-Karls University, Tuebingen, Germany; Tallinn University of Technology, ESTONIA

## Abstract

Surfaces with regular contact with the human body are typically contaminated with microorganisms and might be considered as fomites. Despite spectacles being widespread across populations, little is known about their microbial contamination. Therefore, we swab-sampled 11 worn spectacles within a university setting as well as 10 worn spectacles in a nursing home setting. The microbial load was determined by aerobic cultivation. All spectacles were found to be contaminated with bacteria, with nose pads and ear clips having the highest density, i.e. at sites with direct skin contact. Summed over all sites, the median microbial load of the university spectacles (1.4 ± 10.7 x 10^3^ CFU cm^-2^) did not differ significantly from the spectacles tested in the nursing home (20.8 ± 39.9 x 10^3^ CFU cm^-2^). 215 dominant bacterial morphotypes were analyzed by MALDI biotyping. 182 isolates could be assigned to 10 genera, with *Staphylococcus* being the most common. On genus-level, bacterial diversity was greater on nursing home spectacles (10 genera) compared to the university environment (2 genera). Four cleaning methods were investigated using lenses artificially contaminated with *Escherichia coli*, *Micrococcus luteus*, a 1:2 mixture of *E*. *coli* and *M*. *luteus*, and *Staphylococcus epidermidis* (the dominant isolate in our study), respectively. Best cleaning results (99% -100% median germ reduction) were obtained using impregnated wipes; dry cleaning was less effective (85% -90% median germ reduction). Finally, 10 additional worn university spectacles were cleaned with wipes impregnated with an alcohol-free cleaning solution before sampling. The average bacterial load was significantly lower (0.09 ± 0.49 x 10^3^ CFU cm^-2^) compared to the uncleaned university spectacles previously investigated. Spectacles are significantly contaminated with bacteria of mostly human skin origin—including significant amounts of potentially pathogenic ones and may contribute to eye infections as well as fomites in clinical environments.

## Introduction

The human body is colonized by approximately 10^13^ microorganisms. Cell densities on the human skin can vary from 10^2^ cm^-2^ up to 10^6^ cm^-2^ [1, 2]. Therefore, surfaces regularly touched by humans or those in close contact with the human body can consequently become contaminated with microorganisms and these surfaces can be considered fomites. For instance, mobile communication devices and the touchscreens of computers, tablets, and smartphones are notorious for contributing to fomites in clinical environments [3, 4]. Recently, we reported the ambient bacterial load of smartphone touchscreens from a non-clinical university environment [5]. Uncleaned touchscreens were just moderately (1.37 CFU (colony forming unit) cm^-2^) contaminated with bacteria of mostly human origin, including significant amounts of potentially pathogenic ones. Cleaning with alcohol-impregnated lens wipes effectively reduced bacterial contamination by 96%, thereby lowering any potential risk of infection.

Spectacles are globally widespread optical devices that aid human vision. Due to their environmentally exposed position in the center of the human face, their close contact to the human skin, nose and mouth and regular contact with human hands, it is safe to assume that spectacles are contaminated with microorganisms. Thus far, only a few studies have analyzed the microflora of spectacles. In clinical environments, surgeons’ spectacles were identified as fomites [6]. Their spectacles were highly contaminated with *Staphylococcus epidermidis* and it has been suggested that this represents a risk to patients during operations. Consequently, it was recommended that surgeons disinfect their spectacles on a regular basis. Another recent study [7] addressed the microbiological safety of glasses distributed at 3D movie theatres and the study compared manual vs. automated sanitation systems. The spectacles under investigation were discovered to be contaminated with bacteria and fungi, however, the study did not clearly recommend an effective sanitation system.

In this study, we quantified the microbial load of 31 worn spectacles at 7 different sampling sites, each, and subsequently identified the dominant bacteria. 11 spectacles were obtained from a university environment, 10 spectacles from a nursing home and another 10 spectacles were obtained from the university environment, but were cleaned prior to investigation. In effect, we analyzed spectacles from two different populations–from students and employees at a non-clinical university environment and from inhabitants of a local nursing home. Moreover, we investigated the antimicrobial efficacy of 4 widespread spectacle cleaning methods by using test bacteria that had been previously identified as being dominant on spectacles and smartphone touchscreens. To the best of our knowledge, this is the most comprehensive study on the microflora of spectacles thus far. This study intends to create a solid basis that will invite a deeper understanding of the hygienic relevance of these widespread objects and of the evaluation of suitable cleaning and disinfection measures.

## Material and methods

### Microbial load of worn spectacles

The sampling of worn spectacles was performed in Villingen-Schwenningen, Baden-Württemberg, Germany during the summer of 2015. The spectacles for swab-sampling were kindly provided by 11 students and employees (mean age ± standard deviation: 43.8 ± 19.8 a, 8 females and 3 males) of Furtwangen University, Campus Villingen-Schwenningen, and 10 inhabitants of a local nursing home (mean age: 89.6 ± 6.3 a, 10 females). Spectacles and usage data were provided voluntarily. Informed consent to use the obtained data for scientific purposes was obtained orally. The sampling done in the nursing home was communicated and supported by the directorate of the institution. Personal data of the participants was not recorded, rendering it impossible to assign spectacle microbiota to a specific wearer. Moreover, the spectacle wearers provided neither directly health-related data, nor were the analyses aimed at detecting directly health-related bacteria, such as obligate pathogens or MRSA. Therefore, we believe that the study was performed in an ethically appropriate manner.

Sampling was performed in the field, i.e. within the university and nursing home. Each pair of spectacles was sampled at 7 sites: lenses (left and right, front side and back side, respectively), ear clips (left and right side, respectively) and nose pads. The area of each sampled site was calculated by measuring the geometry of the spectacles. Microbial loads were determined according to DIN 10113–1:1997–07 –Part 1 [8]. Surfaces were meander sampled using sterile cotton swabs (Deltalab, Barcelona, Spain). Each area was sampled twice, first with a wet swab and then with a dry swab. A sterilized medium (wetting medium) containing 1.5 g of casein peptone (Carl Roth GmbH & Co. KG, Karlsruhe, Germany) and 12.75 g of sodium chloride (Carl Roth) per 1500 ml of water was used for wetting the swabs and for subsequent dilution series. After sampling, wet and dry swab heads were combined and microorganisms were extracted by rigorous shaking in a defined volume of wetting medium. The volume of wetting medium used for extraction depended on the sampled area: 2 ml of medium were used per 1 cm^-2^. Germ numbers were determined from that suspension by serial decadal dilution and subsequent plating of 50 μl of each dilution on Tryptic Soy Agar with neutralizers (TSA; Carl Roth). Germ numbers were determined after aerobic incubation at 37°C for 3 d, referred to the sampled area, and then expressed as colony-forming units (CFU) cm^-2^.

### Identification of microbial isolates by MALDI biotyping

In order to get an overview of the microbial diversity on worn spectacles, a representative of each microbial morphotype was isolated per sampling site and spectacle, respectively, i.e. from the agar plates used for quantification of the microbial load. Morphotypes were visually differentiated based on colony size, color and morphology. Selected colonies were repeatedly T-streaked on TSA-Agar, cultivated at 37°C, and then controlled for morphotype purity. A colony of each overnight culture was then suspended in 300 μl of sterile water and stored at -80°C until further analysis.

The identification of isolated microorganisms was performed by means of matrix-assisted laser desorption/ionization time of flight (MALDI-TOF) analysis using the MALDI Biotyper system (MALDI Biotyper Microflex, Bruker Daltonics GmbH, Bremen, Germany) according to the manufacturer’s instructions. One day prior to the analysis, proteins were extracted from the frozen colony samples following the recommended protocol for ethanol-formic acid extraction [9]. The volumes of formic acid (Carl Roth) and acetonitrile (Carl Roth) were adapted as specified in the protocol for single, small colonies. Protein extracts were stored at 4°C until further analysis. Subsequently, 0.7 μl of each protein extract was spotted onto the Biotyper steel target. After air drying, the samples were covered with 1 μl of alpha-cyano-4-hydroxycinnamic acid (Bruker Daltonics), air dried again, and then measured. The fingerprint profiles that were obtained were matched against the internal MALDI Biotyper reference library (software version 3.3.1.0, 4613 entries). Similarities were expressed as score values ranging from 0.0–3.0. According to the manufacturer, scores ≥ 1.7 indicate secure genus identification, scores ≥ 2.0 indicate secure genus and probable species identification.

### Standardized cleaning tests

Cleaning tests were performed with *Escherichia coli* K12 (DSMZ 498), *Micrococcus luteus* (DSMZ 1605) and *Staphylococcus epidermidis* (DSMZ 20044) as test bacteria, obtained from the Leibniz Institute DSMZ (DSMZ—German Collection of Microorganisms and Cell Cultures, Braunschweig, Germany). Bacteria were grown aerobically in liquid Lysogeny Broth (LB, Carl Roth). To determine the viable count, bacteria were decadal diluted in wetting medium. 50 μl of each suspension were plated on TSA-Agar with neutralizers and then were incubated aerobically at 37°C. For further analyses, cells were adjusted to densities of 4 x 10^8^ CFU ml^-1^ (*E*. *coli*, *S*. *epidermidis*) and 4 x 10^10^ CFU ml^-1^ (*M*. *luteus*), respectively. *E*. *coli* and *M*. *luteus* were also used in a 1:2 mixture of these cell suspensions.

Test lenses typically used for spectacles (CR39 Index 1.5 with LotuTec Coating) were provided by Carl Zeiss Vision International GmbH (Aalen, Germany). Before and after microbiological testing, lenses were sterilized with 70% ethanol and a 20 min UV-C-treatment (253.7 nm).

All cleaning tests were performed under a laminar flow workbench. Lenses were fully submerged in the bacterial test suspensions for 1 minute and then dried for 30 minutes at room temperature. Subsequently, one half of each glass was cleaned while the other half of the glass remaining uncleaned. Standardized cleaning was performed by wiping the contaminated side of the glass five times up and five times down. 4 different cleaning products, representing widely used spectacle cleaning methods were investigated: 1. Cellulose-based, alcoholic lens cleaning wipes impregnated with ethanol and isopropanol (AN); 2. Cellulose-based, alcohol-free lens cleaning wipes impregnated with an amine-containing cleaning solution (A); 3. Dry cellulose-tissues without a cleaning solution (C); 4. Dry, fine-grained microfabric clothes (M). All cleaning products were supplied by Carl Zeiss Vision International GmbH. 1 minute after cleaning, swab sampling and quantification of bacteria from the cleaned and uncleaned glass sides was performed as described above. Cleaning tests were repeated 10 times for each test bacterium and each cleaning product, respectively.

In order to investigate the effect of cleaning naturally contaminated spectacles, 10 worn spectacles of students and employees (mean age: 40.6 ± 16.1 a) from a university environment were cleaned intensively by wiping the frames, nose pads and lenses with alcohol-free lens cleaning wipes (A). Subsequently, the microbial load was determined using the method described above.

### Statistical analyses

All microbial load data were expressed in the median ± interquartile range (IQR, between the 25% and 75% quartile). Statistical analyses (plots and statistical tests) were performed using R 3.4.1 [10]. Cleaning tests were examined for statistical significance between the microbial load of the cleaned and the uncleaned glass sides using the Wilcoxon signed rank test for differences between paired samples. The antimicrobial efficiency of different cleaning products and the microbial load of worn (and cleaned) spectacles of university members and nursing home inhabitants were compared using Wilcoxon-Mann-Whitney-U tests for independent samples. All tests were two-sided and p-values < 0.05 were considered to represent significant results. No adjustment of p-values for multiple testing was performed as the study was considered an exploratory pilot study.

## Results and discussion

The aim of this study was to investigate site-dependent microbial loads on worn spectacles using cultivation-based techniques. In addition, we investigated four different cleaning techniques for their efficacy in reducing microbial loads on spectacle lenses.

### Microbial load of worn spectacles

While many related studies from (non-) clinical and healthcare environments report diverse contamination rates of, for instance, mobile communication devices [3, 4, 6], quantitative data on the microbial load of spectacles are scarce. For the first time, our study provides a comprehensive examination of spectacle microbiota by aerobic cultivation. We swab-sampled twenty-one spectacles of adult university students or staff (n = 11) and nursing home residents (n = 10).

Bacteriological analyses indicated that all spectacles were contaminated by bacteria. Summed across all sample sites, we determined a median microbial load of 1.4 ± 10.7 x 10^3^ CFU cm^-2^ found on the university spectacles and a median of 20.8 ± 39.9 x 10^3^ CFU cm^-2^ on the nursing home spectacles.

**Fig 1:**
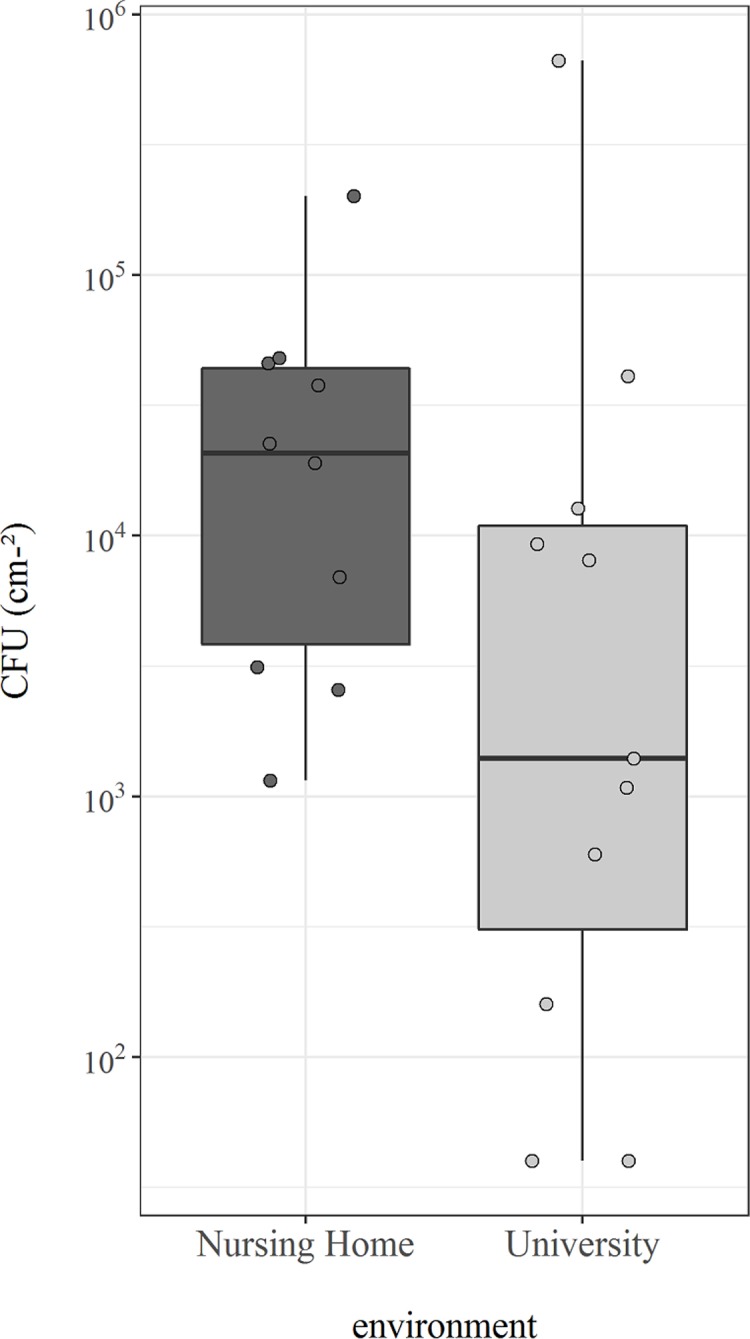
Box-whisker plot showing the determined microbial counts (CFU cm^-2^) of the two investigated environments (nursing home, n = 10, and university, n = 11). Displayed are the median, 25% and 75% quartiles and outliers (open circles). Whiskers represent the lowest and highest microbial counts within the 1.5 fold of the interquartile range (IQR) (the 25% and 75% quartile). An observation is marked as an outlier if it was more than 1.5 times of the IQR away from the 25% or 75% quartile, respectively.

We did not find a statistically significant difference between the microbial loads of the two investigated environments (p = 0.0821; Fig 1), thereby confirming findings of Leyden and colleagues [11], whom did not find differences between the quantity of aerobic and anaerobic bacteria found on the foreheads and cheeks of adults and elderly people.

In both environments, the highest cell counts were found on the nosepads (median of 0.16 ± 2.0 x 10^3^ CFU cm^-2^ on the university spectacles and a median of 2.6 ± 14.1 x 10^3^ CFU cm^-2^ on the nursing home spectacles) while the lowest cell counts were measured on the lenses (median of 0.04 ± 0.08 x 10^3^ CFU cm^-2^ on the university spectacles and a median of 0.23 ± 0.28 x 10^3^ CFU cm^-2^ on the nursing home spectacles, with a significant statistical difference: p = 0,015). This finding matches our assumption that lenses may be more contaminated in the nursing home environment than in a university setting. Due to potentially impaired vision, elderly people may clean the lenses of their spectacle less often or less effectively than younger people with potentially better vision.

Recent studies [12–14] showed that the area behind the ears (retroauricular crease), the sides of the nostrils (alar crease) and the forehead represent the highest density of microbial colonization on the human face. The detected colony counts on nosepads and earclips were indeed similar to colony counts typically found on the human forehead (10^3^ CFU cm^2-^) and the scalp and cheek (about 10^4^ CFU cm^2-^) [15]. Cell densities on spectacle lenses were low in comparison to cell densities on hands, skin, earclips and nosepads. This low count could be due to the smoothness of the lens surfaces, regular cleaning measures, as well as distance to the facial skin.

### Identification of microbial isolates by MALDI biotyping

**Fig 2:**
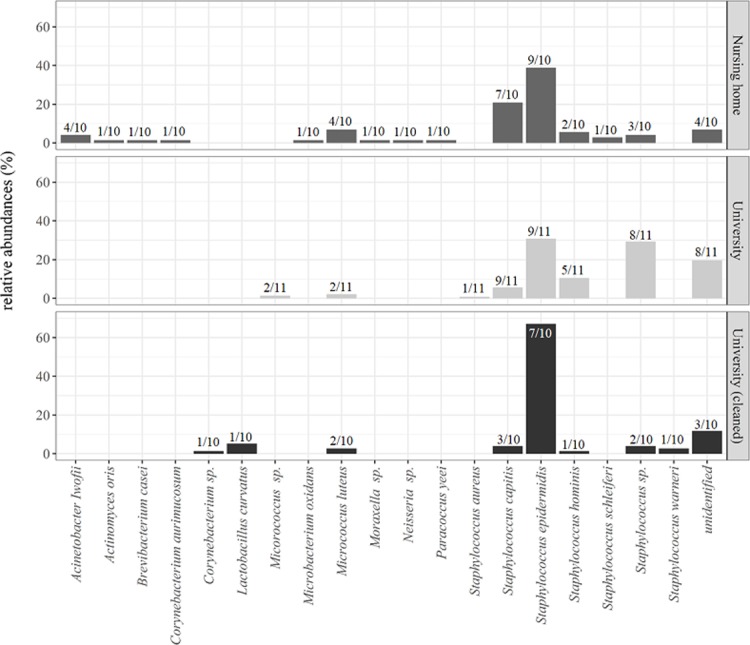
Barplot of identified bacterial taxa isolated from spectacles of two environments. Bars show the relative abundance of isolates from university spectacles (n = 11, 143 isolates), nursing home spectacles (n = 10, 72 isolates) and cleaned university spectacles (n = 10, 76 isolates).”Unidentified” indicates a MALDI identification score below 1.7, where a reliable identification of genus-level was not made possible. Numbers (x/n) on top of the bars indicate the number of spectacles that the respective taxon was detected on.

MALDI-TOF fingerprints of 215 dominant bacterial isolates from all spectacles were generated by MALDI biotyping and used for species and genus-level differentiation. 182 out of 215 isolates could be securely assigned to 10 genera and 12 species. We found a higher number of genera on the spectacles of nursing home inhabitants (10 genera) than on those of the university members (2 genera). Previous studies [16] showed an effect of age on microbial community structure and richness on forehead and scalp, with higher diversity found on elderly individuals. This finding could be based on changes in hormone balances, pH value, and the sebum production [2, 16, 17]. Fig 2 shows the identified genera and the relative abundance of affiliated isolates.

The observed taxa mainly represent well-known colonizers of the human skin, such as staphylococci, micrococci, corynebacteria, brevibacteria or *Acinetobacter* sp. [18]. Staphylococci dominated the aerobic, cultivable spectacles microbiota with *Staphylococcus epidermidis* being the most common representative. Studies [19] have shown that skin areas with direct contact to spectacles, such as the retroauricular crease or the alar crease, are mainly colonized by corynebacteria and propionibacteria and are only sparsely colonized by staphylococci. Given that spectacles represent an aerobic environment, our study was performed under aerobic cultivation conditions. Aerotolerant anaerobes will likely not thrive well under such conditions [20]. Consequently, we did not detect any propionibacteria and we only detected a single isolate of *Corynebacterium* sp. Corynebacteria are aerobic, yet slow growing bacteria, and may have been outcompeted by staphylococci under the used cultivation conditions.

*S*. *epidermidis* is a typical skin commensal, colonizing predominantly the axillae, palms, head and nares [15]. Typically, it does not have strong pathogenic potential, rather, it maintains a commensal or even beneficial relationship with its host. Nevertheless, *S*. *epidermidis* can also lead to severe infections. In addition, it might represent a reservoir for antibiotic resistant genes, which can be transformed into the closely related and more virulent *S*. *aureus* [21]. With *Staphylococcus aureus*, *Staphylococcus epidermidis* and others, we identified many species known to comprise antibiotic resistant strains [22], such as MRSA (*Methicillin-resistant Staphylococcus aureus*) or MRSE (*Methicillin-resistant Staphylococcus epidermidis)*. Hence, further investigations should examine spectacles as carriers of antibiotic resistant bacteria in more detail, an area which could be of particular hygienic relevance in clinical environments.

In order to address the overall pathogenic potential of spectacle microbiota, the identified bacterial species were classified into biosafety risk groups (RG). 60% of the identified bacteria from university spectacles and 64% of the bacteria from nursing home spectacles were affiliated with risk group 2 (RG 2), such as *S*. *epidermidis*, *S*. *hominis*, *S*. *aureus* and *S*. *schleiferi*. The remaining species were classified as non-pathogenic (RG1). RG 2 organisms are harmless for those with intact immune systems but could cause severe diseases for newborns, immunocompromised patients, pregnant women, or elderly persons.

*S*. *epidermis*, *S*. *hominis* and *S*. *aureus* are also related to eye diseases. For instance, both endolphtalmitis and conjunctivitis are often caused by *S*. *epidermidis* [21]. *S*. *hominis*, and *S*. *aureus* had also been associated with intraocular and external ocular infections [23].

Overall, we have shown that spectacles are contaminated with bacteria of mostly human skin origin, including potentially pathogenic ones and should therefore be considered fomites.

### Standardized cleaning tests

Cleaning tests were performed with *Micrococcus luteus* (as a gram positive representative), *Escherichia coli* K12 (as a gram negative representative), a 1:2 mixture of *E*. *coli* and *M*. *luteus* and *Staphylococcus epidermidis*. The latter was chosen as it was the dominant spectacle isolate in our study. Test lenses were artificially contaminated with bacteria and then cleaned using 4 different cleaning procedures.

**Fig 3:**
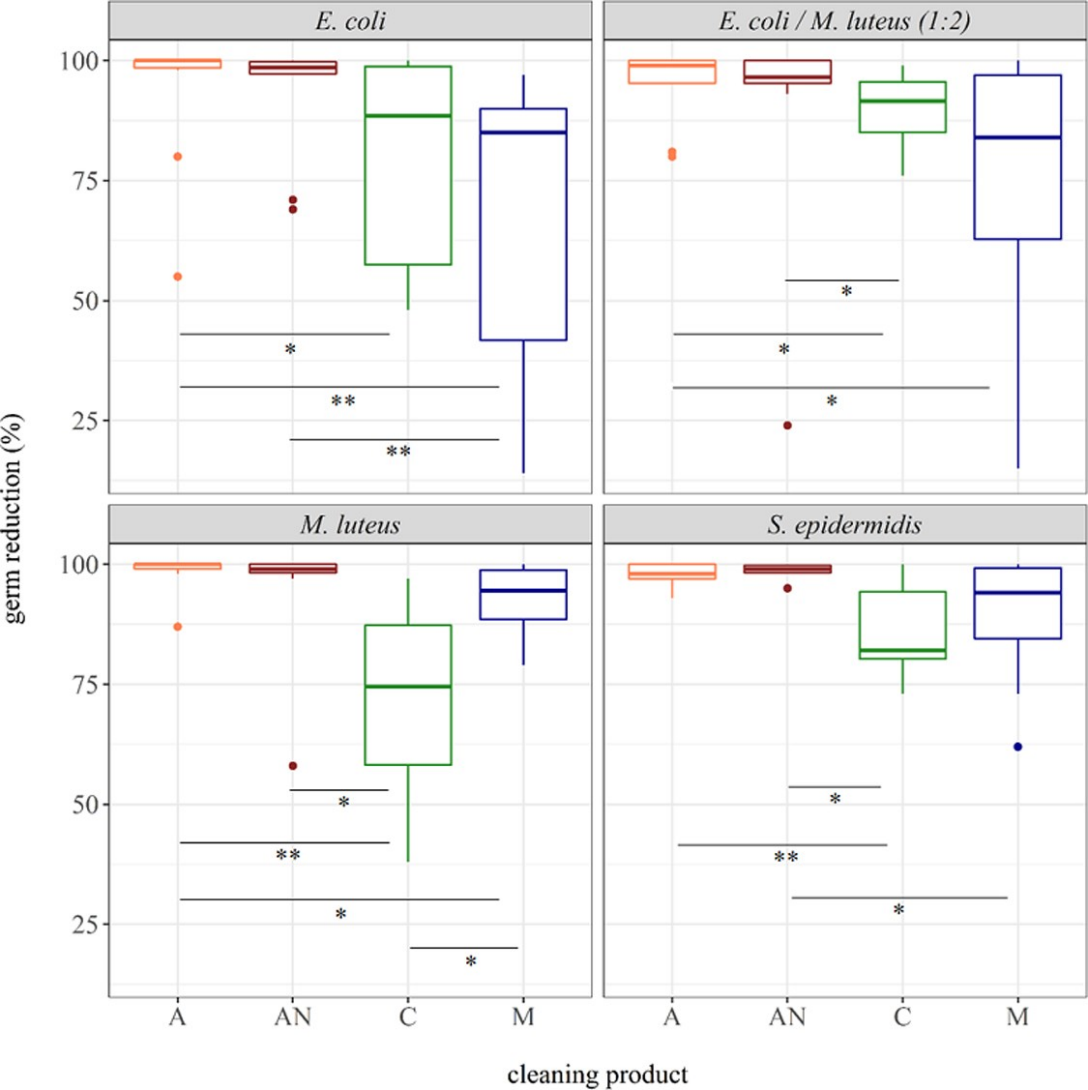
Box-whisker plot showing the relative germ reduction of 4 different cleaning procedures, calculated from the microbial load before and after the cleaning. A (orange) = cellulose-based, alcoholic lens cleaning wipes; AN (red) = cellulose-based, alcohol-free lens wipes, C (green) = dry cellulose-tissue; M (blue) = fine-grained microfabric cloth (n = 10 for all test bacteria and cleaning products, respectively). Displayed are the 25% and 75% quartiles, median and data outliers (open circles). Whiskers represent the lowest and highest microbial counts within the 1.5 fold of the interquartile range (IQR) (the 25% and 75% quartile). An observation is marked as an outlier if it was more than 1.5 times of the IQR away from the 25% or 75% quartile, respectively. Asterisks mark a statistically significant difference between the cleaning products: * p < 0.05; ** p < 0.01.

All tested procedures removed a significant amount of bacteria from lens surfaces (p < 0,001). Fig 3 shows the measured germ reductions.

Across all test bacteria, the impregnated cellulose wipes (A and AN) showed the most germ reduction (means of 99% - 100%). The efficacy of the alcoholic lens wipes (A) was slightly higher when compared to the alcohol-free formulation (AN), however, differences were not significant (p = 0,228–0,746).

Dry cleaning with cellulose tissues (C) and microfabric clothes (M) showed reduced cleaning success (mean germ reduction of 85%– 90%) when compared to cleaning with the impregnated wipes. The observed differences between these products (dry and impregnated) were mostly statistically significant (p < 0.05, p < 0.01, respectively). Evidently, wet cleaning was more effective than pure mechanical cleaning with dry wipes.

The cleaning performance of the alcohol-impregnated wipes is presumably largely based on their ethanol or isopropyl alcohol content, which are well known antimicrobials. Ethanol and isopropyl alcohol destroy the bacterial cell wall and plasma membrane by denaturing proteins and dissolving lipids, leading to subsequent interference with metabolism and cell lysis. Alcoholic formulations represent effective antimicrobial agents against vegetative bacteria, mycoplasms, fungi and viruses [24].

The investigated non-alcoholic lens wipes contain detergents such as alkyl-dimethylamine oxides, which have also been shown to have antimicrobial effects. These substances act as amphoteric surfactants by disrupting and perturbing the bacterial cell membrane [25]. Consequently, these wipes showed similar cleaning efficacy compared to the alcohol impregnated wipes. However, using non-alcoholic products to clean spectacles may be favorable so as to protect sensitive parts of the spectacles, such as the frame material, from damage.

In order to verify the measured germ reduction for naturally contaminated spectacles, 10 worn spectacles from university staff and students were, prior to swab-sampling, thoroughly cleaned using wipes impregnated with the alcohol-free cleaning solution. Following cleaning, 3 spectacles showed no bacterial contamination at all, 4 spectacles showed slight contamination on the nosepads and earclips and 3 spectacles showed germs on the lenses. Compared to the total germ count of uncleaned spectacles from university members (see above), we calculated 94% less bacteria on the cleaned spectacles (median 0,09 ± 0.49 x 10^3^ CFU cm^-2^). In addition, we identified less but largely the same species, on university and nursing home spectacles. We also found two additional species, *Lactobacillus curvatus* and *Staphylococcus warner*i, which also represent typical human skin commensals (Fig 2).

## Conclusion

Our results provide the first insight into the aerobic and cultivable spectacle microbiota. All investigated worn spectacles were found to be contaminated by bacteria of mostly human skin origin, in particular at sites with direct skin contact. The bacterial community was highly dominated by staphylococci, in particular *S*. *epidermidis*. No propionibacteria were found, which is likely due to the aerobic cultivation conditions. Many of the identified bacteria represented potential pathogens and some of them are known to cause skin and eye diseases. Hence, spectacles should certainly be seen as fomites, particularly in clinical environments where transmission of pathogens could occur through spectacle contamination. In addition, spectacles could represent a reservoir for pathogens causing recurring eye infections. However, we also demonstrated that superficial cleaning with impregnated lens wipes can reduce microbial load by ~ 2 log scales and thus help prevent bacterial transfer.

For future investigation we will conduct 16S rRNA gene next generation sequencing based analyses of spectacle microbiota in order to better account for aerotolerant anaerobic, slow-growing and yet-uncultured microorganisms. In addition, particular emphasis will be placed on spectacles as carriers of multi-resistant bacteria in clinical and nursing environments.

## Supporting information

S1 Table Germ counts of the spectacles at the different sample sites and environments. (XLSX)

S2 Table Number of isolates from the spectacles and the identified bacterial taxa. (XLSX)

S3 Table Germ counts of the cleaning tests. (XLSX)
